# Supplementary material for: In situ grown oxygen-vacancy-rich copper oxide nanosheets on a copper foam electrode afford the selective oxidation of alcohols to value-added chemicals
Source: Commun Chem. 2022 Sep 12;5:109. doi: 10.1038/s42004-022-00708-1 (PMC9814762; doi:10.1038/s42004-022-00708-1)
Supplement: Supplementary file 2 — Supplementary Information [file 42004_2022_708_MOESM2_ESM.pdf]

In situ grown oxygen-vacancy-rich copper oxide nanosheets on a copper foam electrode afford the selective oxidation of alcohols to value-added chemicals

Mustafa Khan[1], Asima Hameed[1], Abdus Samad[2], Talifhani Mushiana[1], Muhammad Imran Abdullah[3]*, Asma Akhtar[1], Raja Shahid Ashraf[3], Ning Zhang[4], Bruno G. Pollet[5], Udo Schwingenschlögl[2]*& Mingming Ma[1]*

1 Hefei National Laboratory for Physical Sciences at the Microscale, Department of Chemistry, University of Science and Technology of China, Hefei, Anhui 230026, China

2 Physical Science and Engineering Division, King Abdullah University of Science and Technology (KAUST), Thuwal 23955-6900, Saudi Arabia

3 Department of Chemistry, Government College University Lahore, 54000, Pakistan

4 Department of Biology and Environmental Engineering, Hefei University, Hefei, Anhui 230022, China

5 Pollet Research Group, Hydrogen Research Institute (HRI), Université du Québec à Trois-Rivières, 3351 Boulevard des Forges, Trois-Rivières, Québec G9A 5H7, Canada

E-mail: udo.schwingenschlogl@kaust.edu.sa; mma@ustc.edu.cn; mimran@mail.ustc.edu.cn

Table of Contents

1. Supplementary Note 1 3
2. Supplementary Note 2 4
3. Supplementary Note 3 7
4. Supplementary Note 4 9
5. Supplementary Notes 5........ 11
6. Supplementary Note 6 13
7. Supplementary Note 7 14
8. Supplementary Note 8 19
9. Supplementary Table 1. 21
10. Supplementary Table 2. 21
11. Supplementary Table 3. 24
12. Supplementary References........ 25

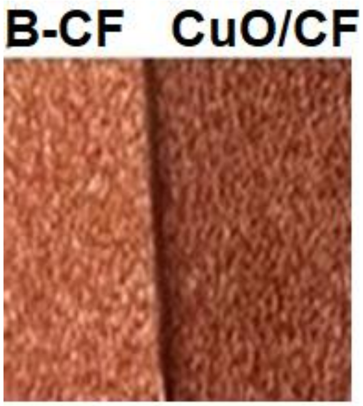

Supplementary Figure 1. Photographs of the bare CF (B-CF) and as-prepared CuO/CF.

1. Supplementary Note 1

The liquid products were analyzed by nuclear magnetic resonance (NMR) spectrometer Bruker -500 NMR. The products of MOR and EOR (0.5 mL electrolyte was added to 0.1 mL D_2O) were identified and quantified by ^{13}CNMR and ^{1}HNMR experiments; a pronounced chemical shift at 171.1 ppm associated with formate was detected in ^{13}CNMR spectra of all tested samples see Supplementary Figures 16b, and 17b. Similarly, the chemical shifts around 181 ppm correspond to acetate after EOR as presented in Supplementary Figures 16d and 17d. Quantitative analysis of the formate and acetate formed during MOR and EOR were carried out with ^{1}HNMR, where maleic acid (0.1 mL) was used as an internal standard for the quantification of both products. A vibrant chemical shift around 8 ppm corresponding to format was found in all samples analyzed after MOR, while the ^{1}HNMR peak for an internal standard appears around 6 ppm as shown in Supplementary Figures 16a and 17a. Likewise, the ^{1}HNMR peak around 2 ppm belongs to acetate formed during EOR see Supplementary Figure 16c and 17c. The corresponding Faraday efficiencies for formate and acetate were calculated at different potentials. Moreover, the yield (%) and selectivity (%) of products for each reaction were calculated at a static potential for different durations with the following equations.

$$\text{Yield}(\%) = \frac{\text{moles of formed product}}{\text{initial moles of reactant}} \times 100 \quad (1)$$

$$\text{Selectivity}(\%) = \frac{\text{mole of formed product}}{\text{mole of reactant consumed}} \times 100 \quad (2)$$

The Faradaic efficiency (*FE*) for the formation of formate and acetate on CuO/CF as anode were calculated as:

$$FE(\%) = \frac{\text{moles of formed product}}{\text{total passed charge}/(4\times F)} \times 100 \qquad (3)$$

Products = Formate and Acetate

Where *F* is the Faraday constant (96485 C mol^{-1})

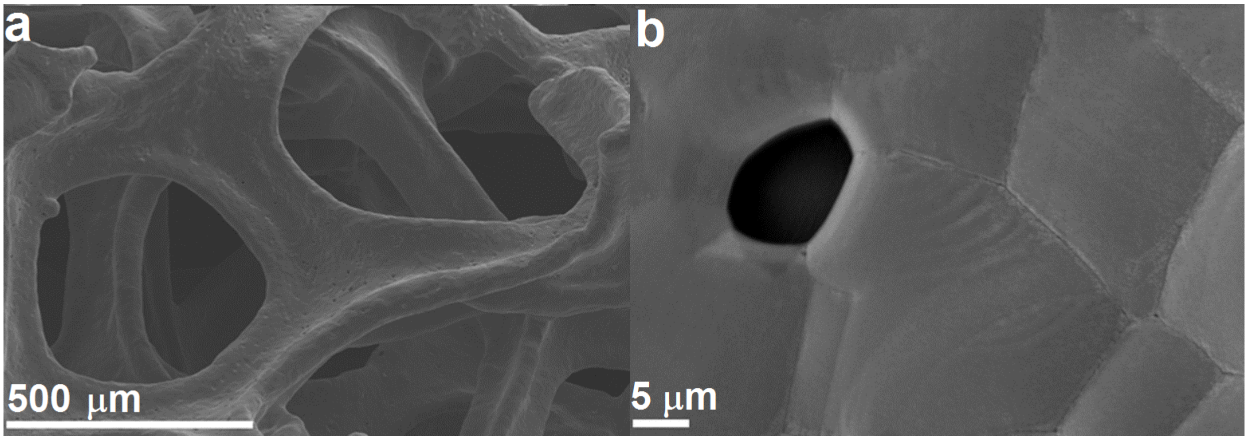

Supplementary Figure 2. a-b) SEM images of B-CF.

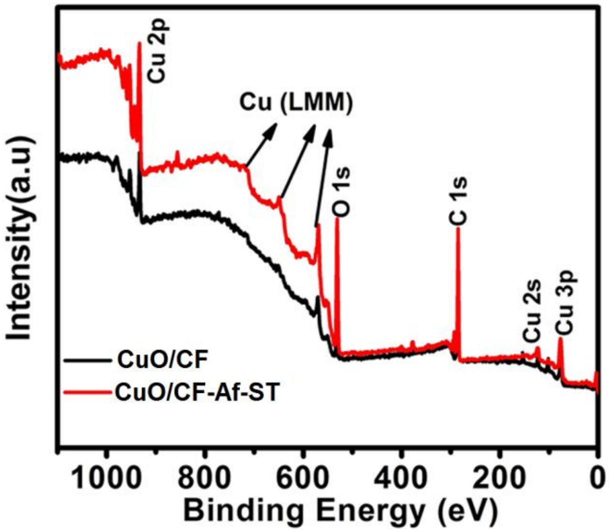

Supplementary Figure 3. XPS survey spectra of the as-prepared CuO/CF before and after the AOR stability test (ST).

2. Supplementary Note 2

As it is very much possible to obtain a misleading electrocatalytic activity while using Ag/AgCl as a reference electrode in alkaline electrolyte. For this purpose, we compare the EOR

electrocatalytic activity for the same electrocatalyst in the same electrolyte with Ag/AgCl and Hg/HgO as reference electrodes. Supplementary Figure 4a, shows the EOR activity of the as-prepared CuO/CF measured in the same electrolyte solution with Ag/AgCl and Hg/HgO as reference electrodes in a potential window ranging from -0.2 to 1 V vs Ag/AgCl or Hg/HgO. Supplementary Figure 4b, displays the EOR data in the reversible hydrogen electrode (RHE) scale obtained with Ag/AgCl and Hg/HgO reference electrodes. As presented the EOR activity obtained for the as-prepared CuO/CF with both Ag/AgCl and Hg/HgO reference electrodes were quite similar. Furthermore, a chronoamperometry (CA) experiment was carried out at an applied potential of 0.8 V *vs* Ag/AgCl for the duration of 11 hours to notice if any possible change in the EOR activity for the as-prepared CuO/CF after the prolonged use of Ag/AgCl in CA test depicted in Supplementary Figure 4c. As displayed in Supplementary Figure 4d, the LSV curves taken before and after CA test were also quite similar which nullifies the possibility of reporting misleading activity in this work. However, to be on the safe side we strictly recommend the use of Hg/HgO as a reference electrode in the alkaline electrolyte.

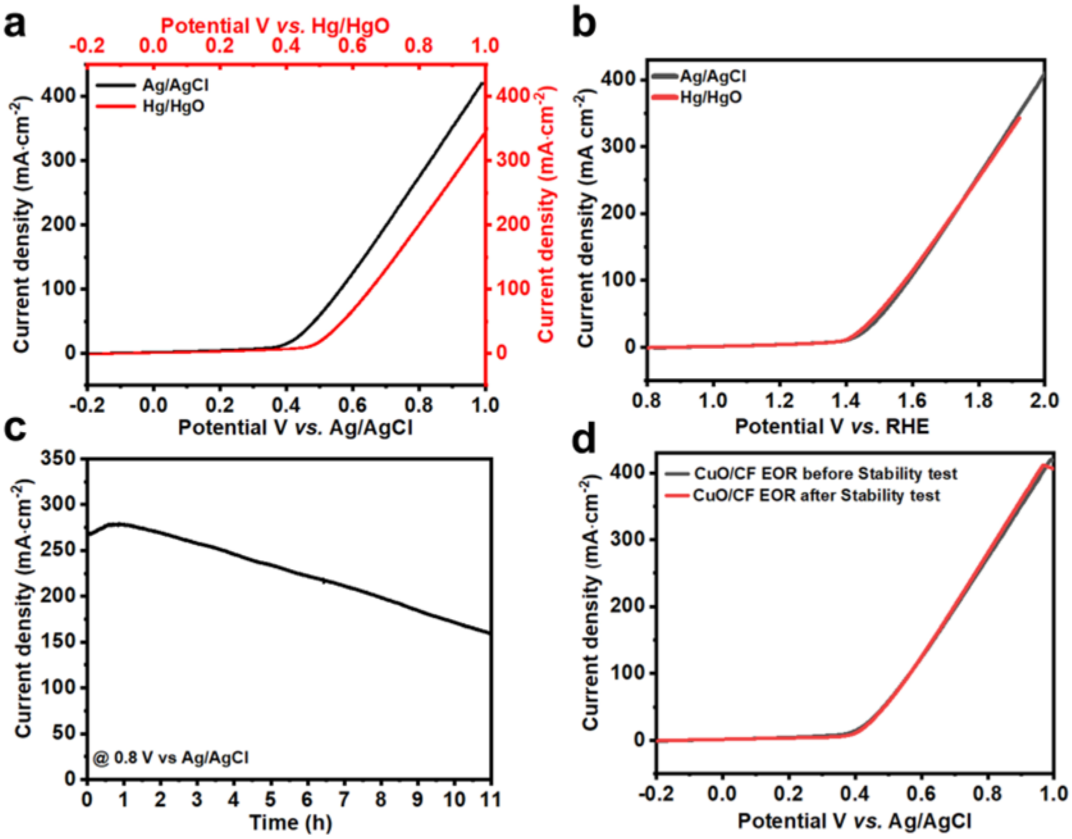

Supplementary Figure 4. Comparative EOR activity of the as-prepared CuO/CF obtained with different reference electrodes. a) EOR activity of the as-prepared CuO/CF obtained with Ag/AgCl and Hg/HgO as reference electrodes. b) EOR activity of the as-prepared CuO/CF obtained with Ag/AgCl and Hg/HgO in Reversible Hydrogen Electrode (RHE) scale. c) Long-term stability test conducted at 0.8 V *vs* Ag/AgCl for 11 hours. d) Comparative LSVs before and after long-term stability test for the as-prepared CuO/CF using Ag/AgCl as reference electrode after being used for 11 hours in stability test.

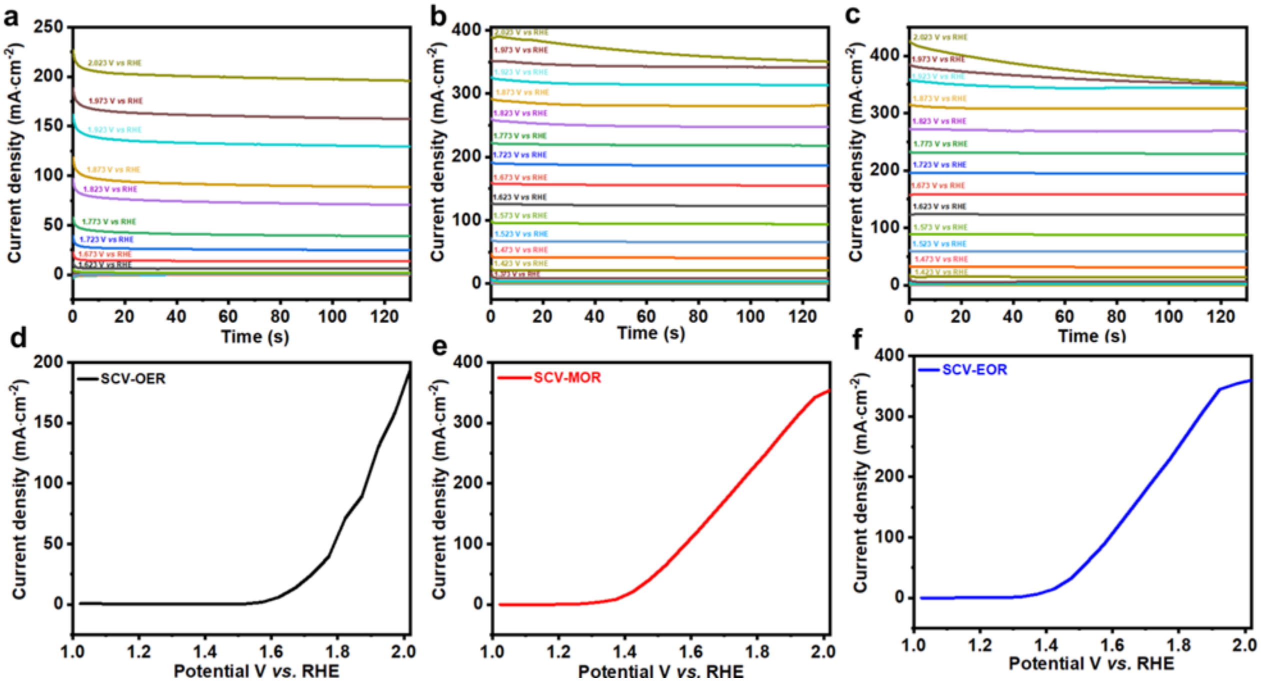

Supplementary Figure 5. Chronoamperometry (CA) response of the as-prepared CuO/CF at constant potential from 1.023 to 2.023 V *vs* RHE. a) Oxygen evolution reaction (OER), b) methanol oxidation reaction (MOR), and c) ethanol oxidation reaction (EOR). Sample Current Voltammetry (SCV) curve derived from the respective CA response obtained at different constant potential for the as-prepared CuO/CF in d) OER, e) MOR, and f) EOR. Note that all the SCV curves were constructed from the current collected at the 100th second at each applied potential.

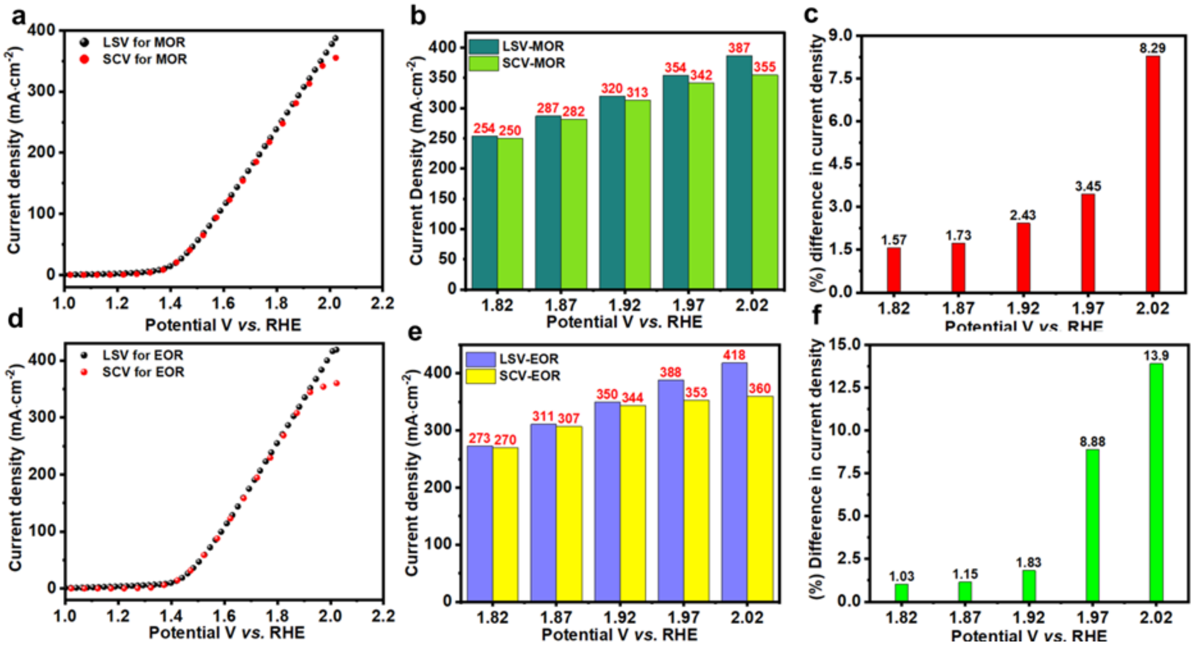

Supplementary Figure 6. Comparative MOR and EOR activity of the as-prepared CuO/CF measured with LSV and SCV. a,d) MOR and EOR data for the as-prepared CuO/CF obtained with LSV (Black dots) and SCV (Red dots). b,e) Bar graphs for MOR and EOR activity obtained with LSV and SCV compared at different potentials. c,f) Summaries of (%) difference in MOR and EOR current densities measured with LSV and SCV at different potentials.

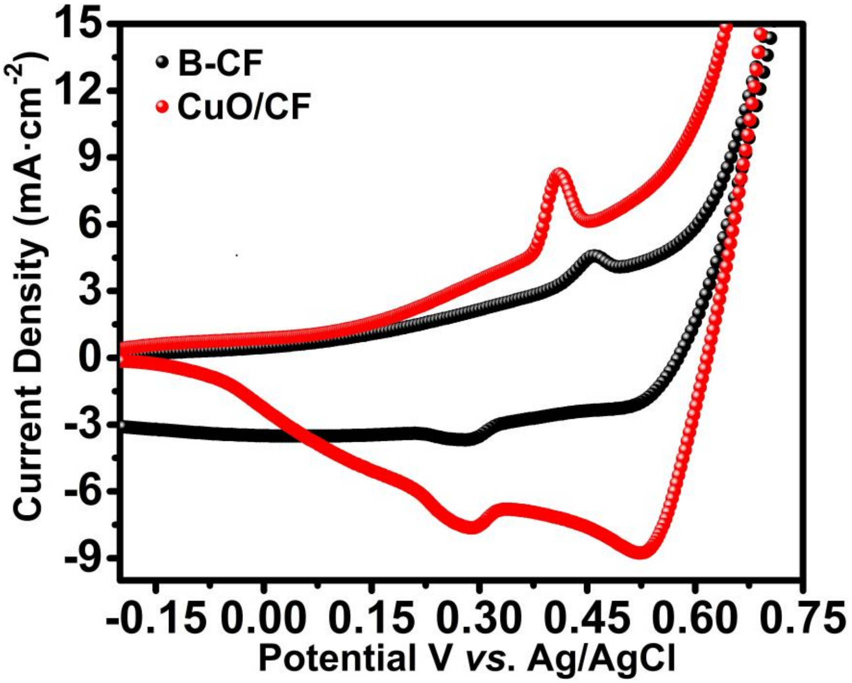

Supplementary Figure 7. CVs of the B-CF and as-prepared CuO/CF electrodes in 1M KOH at a scan rate of 50 $mV \cdot s^{-1}$.

3. Supplementary Note 3

The surface coverage of Cu(II)/Cu(III) redox species in both B-CF and as-prepared CuO/CF were studied based on the linear relation between the peak current density values of the Cu(II)/Cu(III) redox couple and the scan rate using cyclic voltammetry. The cyclic voltammograms for both as-prepared CuO/CF and B-CF were collected in a 1M KOH solution at varied scan rates ranging from 5 to 100 $mV \cdot s^{-1}$. Both the anodic and cathodic peak current densities related to Cu(II)/Cu(III) redox couple increases linearly upon increasing the scan rate. As a result, the surface coverage of the Cu(II)/Cu(III) redox species in the B-CF and as-prepared CuO/CF electrodes can be calculated according to equation 4.[1]

$$I_P = \left(\frac{n^2F^2}{4RT}\right) A\Gamma^* v \quad (4)$$

where I_p, n, F, R, T, v, A, and $\Gamma*$ correspond to peak current density, number of transferred electrons, Faraday constant (96845 C mol^{-1}), ideal gas constant (8.314 $JK^{-1}mol^{-1}$), temperature (298 K), potential scan rate, the geometric surface area of the electrode, and surface coverage of the redox species, respectively. According to the slope of straight lines, the obtained $\Gamma*$ for Cu(II)/Cu(III) redox couple redox was determined by taking the average of both cathodic and anodic current densities, and the estimated values of $\Gamma*$ were found to be 1.43 x 10^{-7} mol·cm^{-2}, for CuO/CF electrode. A good linear relation of the anodic and cathodic peak current densities of the as-prepared CuO/CF electrode to the square root of the scan rate suggests that the total redox transition of Cu(II) is a diffusion-controlled process. [2] It has been reported that the Cu(II)/Cu(III) redox process is controlled by the solid-phase body of the proton diffusion.[1, 3] Therefore, the *D* of the rate-limiting proton is an important parameter for the evaluation of the electrochemical behavior of the electrocatalysts. The value of *D* at 298 K can be estimated with the help of equation 5.

$$I_P = 2.69 \times 10^5 n^{\frac{3}{2}} A D^{\frac{1}{2}} C\, v^{\frac{1}{2}} \quad (5)$$

where I_p, n, A, D, C, and v are the corresponding anodic peak current, number of transferred electrons, geometric area of the electrode, proton diffusion coefficient, proton concentration, and the potential scan rate, respectively. The proton concentration in the copper hydroxide ($Cu(OH)_2$ is assumed to be the same as that of Cu(II) according to the stoichiometry of the reaction is given below.

CuO + H_2O <=> $Cu(OH)_2$

$Cu(OH)_2$ <=> CuOOH + H^+ + e^-

Taking the initial concentration of proton to be 0.048 mol·cm^{-3}[2, 4], the calculated *D* was found to be 2.17 x 10^{-8} $cm^2 \cdot s^{-1}$ for the as-prepared CuO/CF electrode.

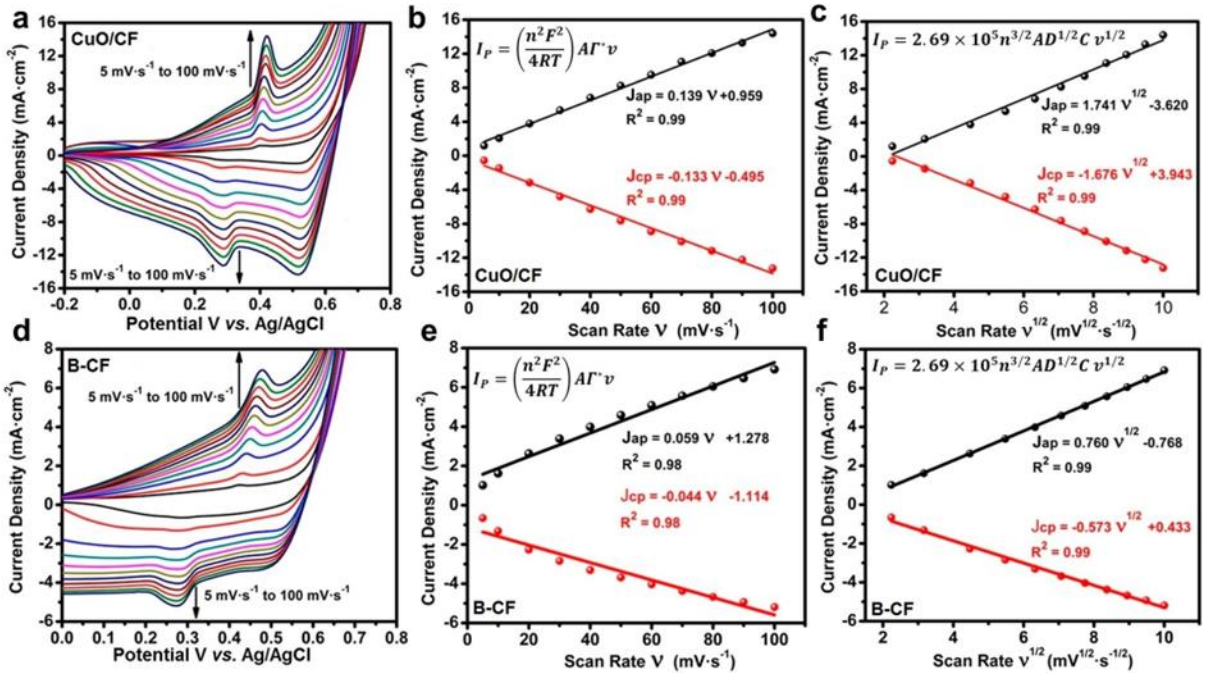

Supplementary Figure 8. a,d) Characteristic CVs of the as-prepared CuO/CF and B-CF electrode in 1M KOH at scan rates of 5, 10, 20, 30, 40, 50, 60, 70, 80, 90, and 100 mV·s^{-1}. b,e) Relationship between the anodic and cathodic peak current densities and the scan rates for as-prepared CuO/CF and B-CF electrodes. c,f) Proportionality of the anodic and cathodic peak current densities to the square roots of scan rates.

4. Supplementary Note 4

As shown in Supplementary Figure 9a-b, both B-CF and as-prepared CuO/CF are active towards MOR and EOR in alkaline electrolytes and exhibit a prominent MOR and EOR current density at lower applied potential compared to OER. However, the as-prepared CuO/CF is more active towards MOR and EOR and has almost double MOR and EOR current densities as compared to the B-CF. Similarly, the EIS results presented in Supplementary Figure 11a-b also confirm the faster MOR and EOR kinetics over the as-prepared CuO/CF. As shown compared to the B-CF, the as-prepared CuO/CF exhibits more depressed semicircles and low charge transfer resistance in alkaline electrolytes containing 1M MeOH or 1M EtOH see Supplementary Figure 10a-b.

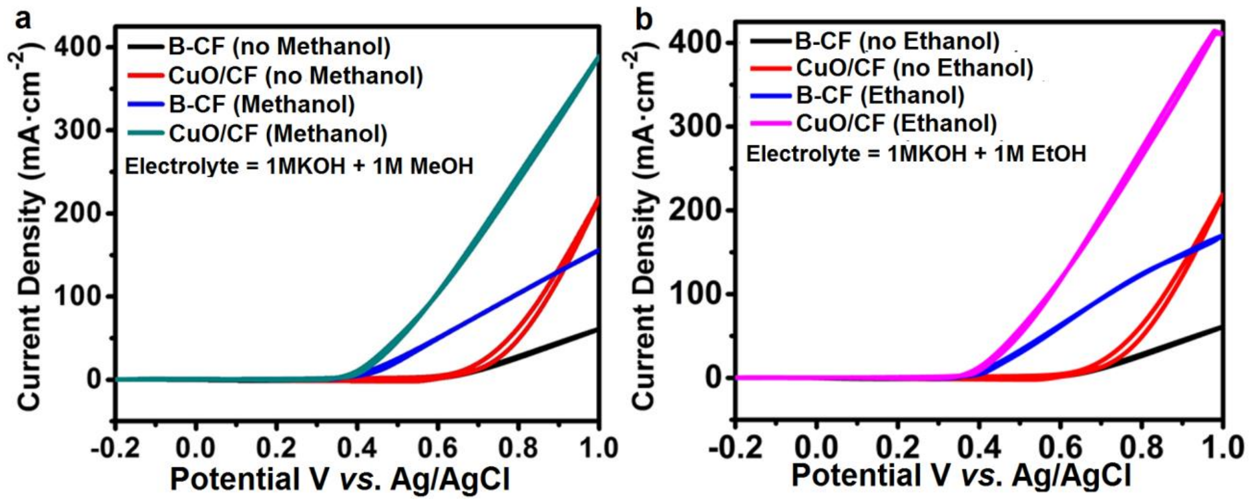

Supplementary Figure 9. a) Cyclic voltammograms of the B-CF and as-prepared CuO/CF in the presence and absence of 1M methanol recorded in 1M KOH at a scan rate of 5 mV·s^{-1}. b) Cyclic voltammograms of the B-CF and as-prepared CuO/CF in the presence and absence of 1M ethanol recorded in 1M KOH at a scan rate of 10 mV·s^{-1}.

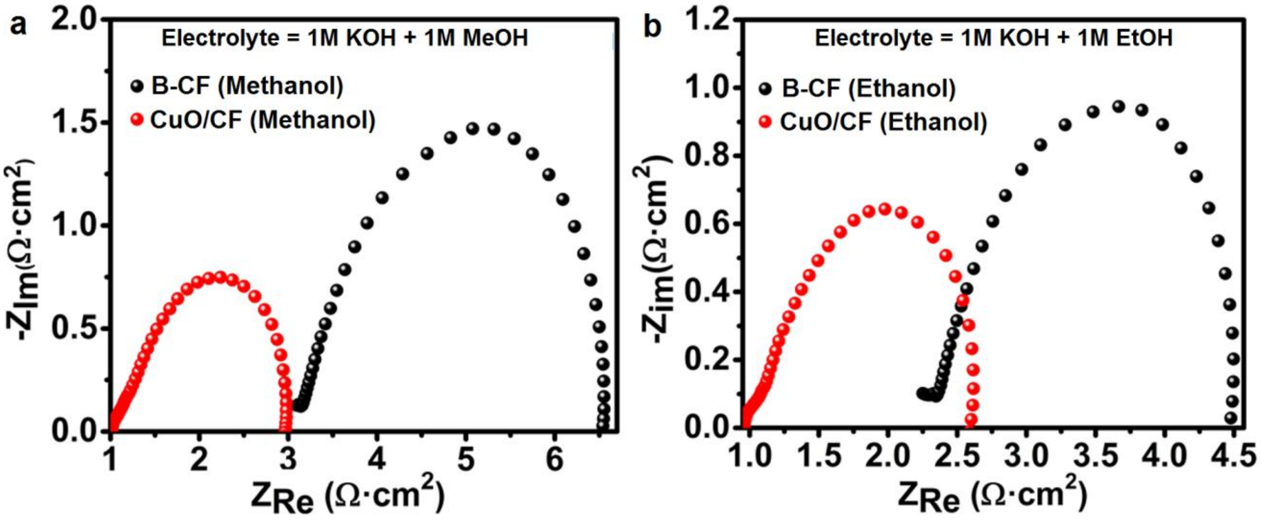

Supplementary Figure 10. a) EIS plots of the B-CF and as-prepared CuO/CF in 1M KOH + 1M MeOH b) EIS plots of the B-CF and as-prepared CuO/CF in 1M KOH + 1M EtOH.

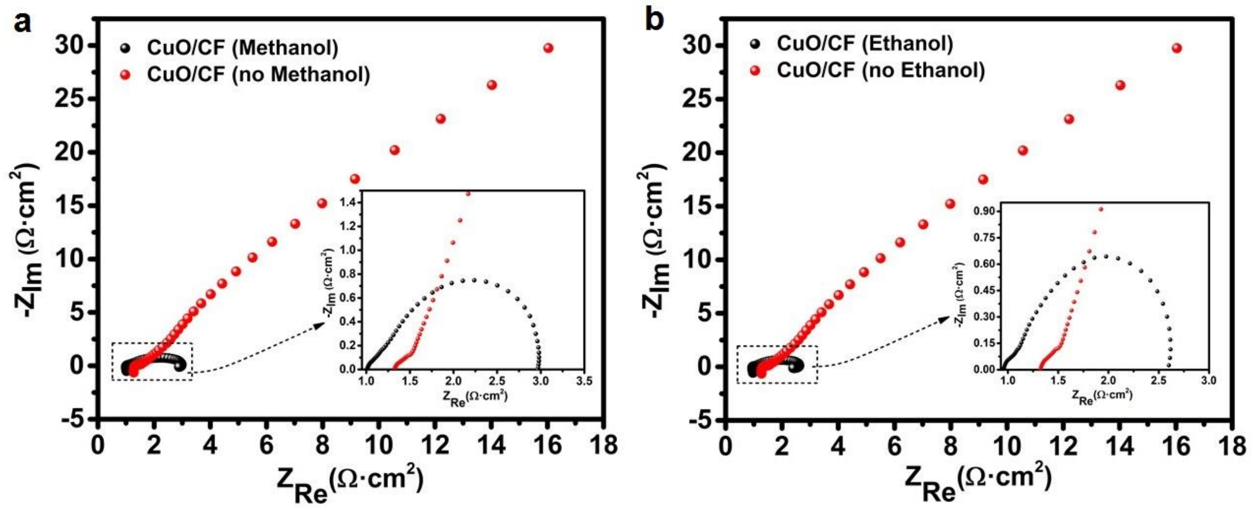

Supplementary Figure 11. a) Nyquist plots of the as-prepared CuO/CF at +0.45 V *vs*. Ag/AgCl with and without 1M methanol in 1M KOH. b) Nyquist plots of the as-prepared CuO/CF at +0.45 V *vs*. Ag/AgCl with and without 1M ethanol in 1M KOH.

5. Supplementary Notes 5

The concentration of EtOH and MeOH is a key variable for EOR and MOR. The effect of EtOH and MeOH concentration over the as-prepared CuO/CF electrodes was investigated in 1M KOH electrolyte see Supplementary Figure 12a,d. As shown, increasing the concentration of EtOH and MeOH from 0.1 to 2M linearly increases the corresponding EOR and MOR current densities. Our results show that the as-prepared CuO/CF can bear higher concentrations of EtOH and MeOH even though the active centers for catalysis become saturated at higher methanol concentrations (>1.5M). Moreover, the EtOH and MeOH oxidation peak current in the anodic sweep was proportionate to the concentration of EtOH and MeOH. The increase in the concentration of EtOH and MeOH produced a closely proportional boost of the methanol and ethanol oxidation peak current density Supplementary Figure 12b,e. The as-prepared CuO/CF has Δj = 196 and 129 $mA \cdot cm^{-2}$ peak current density difference at 2M EtOH and 2M MeOH as compared to 0.1M EtOH and 0.1M MeOH which indicates the robust performance of the as-prepared CuO/CF under high concentration of EtOH and MeOH. Thus, the peak current density for EtOH and MeOH in different concentrations is much higher as compared to the reported electrocatalysts for EOR and MOR see Supplementary Table 2 and Table 3. Furthermore, the R_{CT} of the as-prepared CuO/CF electrode in EOR and MOR reduces as the concentration of EtOH and MeOH increases, indicating that the charge transfer rate was largely reliant on the EtOH and MeOH concentration and oxidation rate on the surface of as-prepared CuO/CF as depicted in Supplementary Figure 13a-b.

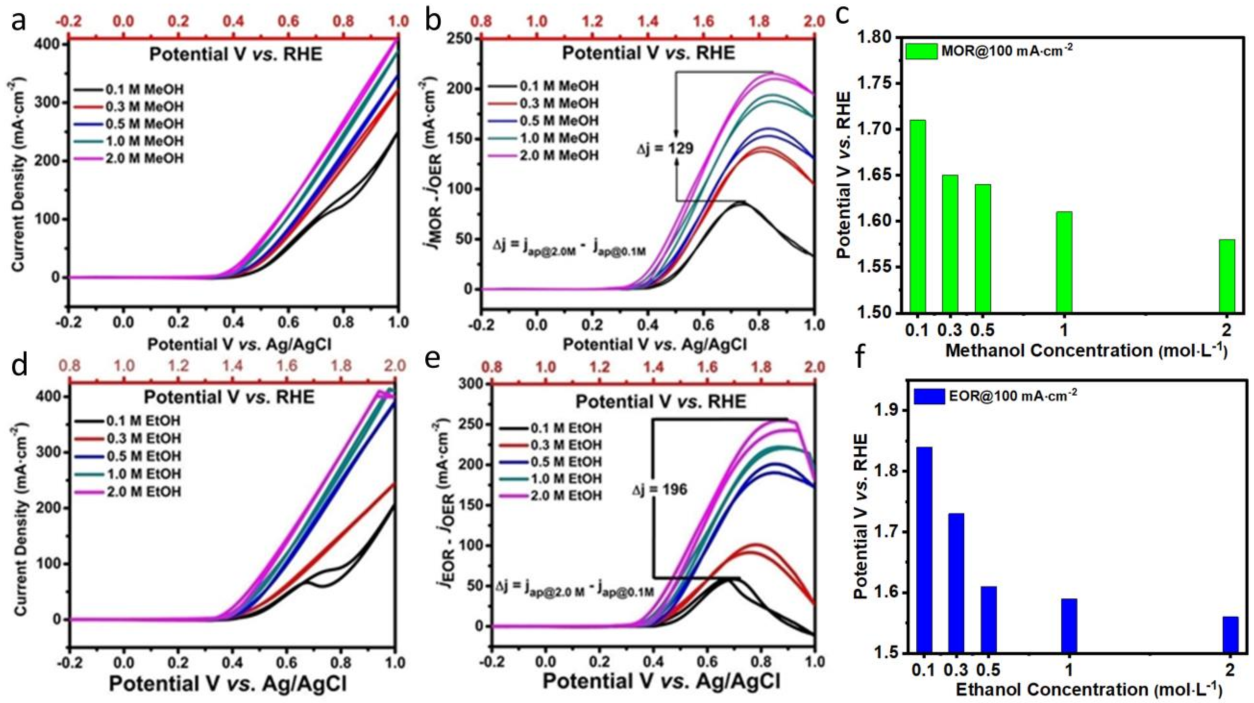

Supplementary Figure 12. a,d) Cyclic voltammograms for the as-prepared CuO/CF in 1M KOH containing different concentrations of methanol or ethanol (0.1, 0.3, 0.5, 1, and 2M) at a scan rate of 10 mV.s^{-1}. b,e) Cyclic voltammograms collected over the as-prepared CuO/CF in 1M KOH solution representing the difference in peak current densities at different concentrations of methanol or ethanol (0.1, 0.3, 0.5, 1, and 2M) at a scan rate of 10 mV·s^{-1}. Here, the baseline current obtained in KOH was subtracted from the overall current. All data were not *iR* corrected. c,d) Summaries of anodic potential to reach 100 mA. cm^{-2} for MOR and EOR at different concentration of methanol and ethanol.

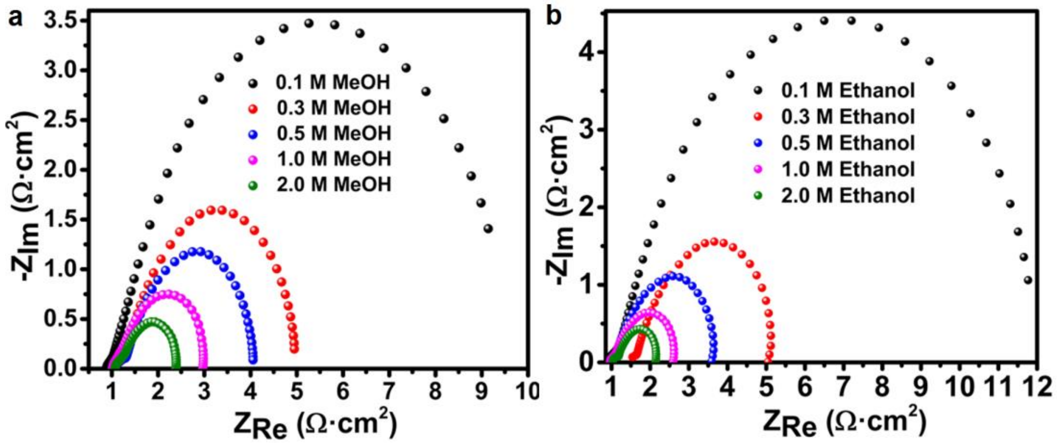

Supplementary Figure 13. a) EIS plots of the as-prepared CuO/CF in 1M KOH solution in the presence of different concentrations of methanol (0.1, 0.3, 0.5, 1, and 2M) b) EIS plots of the as-prepared CuO/CF in 1M KOH solution in the presence of different concentrations of ethanol (0.1, 0.3, 0.5, 1, and 2M).

6. Supplementary Note 6

As displayed in Supplementary Figure 14a-d, the CV curves were collected over the B-CF and as-prepared CuO/CF in 1M KOH containing 1M MeOH or 1M EtOH at different scan rates. As shown, increasing the scan rates does not enhance the current densities in the region where MOR and EOR take place, which reflects that both MOR and EOR are governed by the kinetics of reaction over the surface of the as-prepared CuO/CF, because kinetic current does not increase with increasing scan rate. The inset of Supplementary Figure 14a-d represents the zoomed image of the region where oxidation of Cu^{2+} to Cu^{3+} takes place before MOR and EOR. This observation indicates that the total number of catalytically accessible Cu sites for MOR and EOR was either the Cu^{2+} sites that were oxidized before AOR or only the Cu^{3+} sites that were reduced after the AOR. To reveal the real number of electrochemically accessible Cu sites for the MOR and EOR, a backward scan of the B-CF and as-prepared CuO/CF measured at 200 mV·s^{-1} in 1M KOH containing 1M MeOH or EtOH were used for charge integration and consequent calculations see Supplementary Figure 14e-h. The number of electrochemically accessible Cu atoms in the B-CF and as-prepared CuO/CF were found to be 5.17 × 10^{16}, 5.0× 10^{16} and 6.24 × $10^{16,}$ 7.40 × 10^{16} for MOR and EOR respectively, as presented in Supplementary Figure 14e-h.

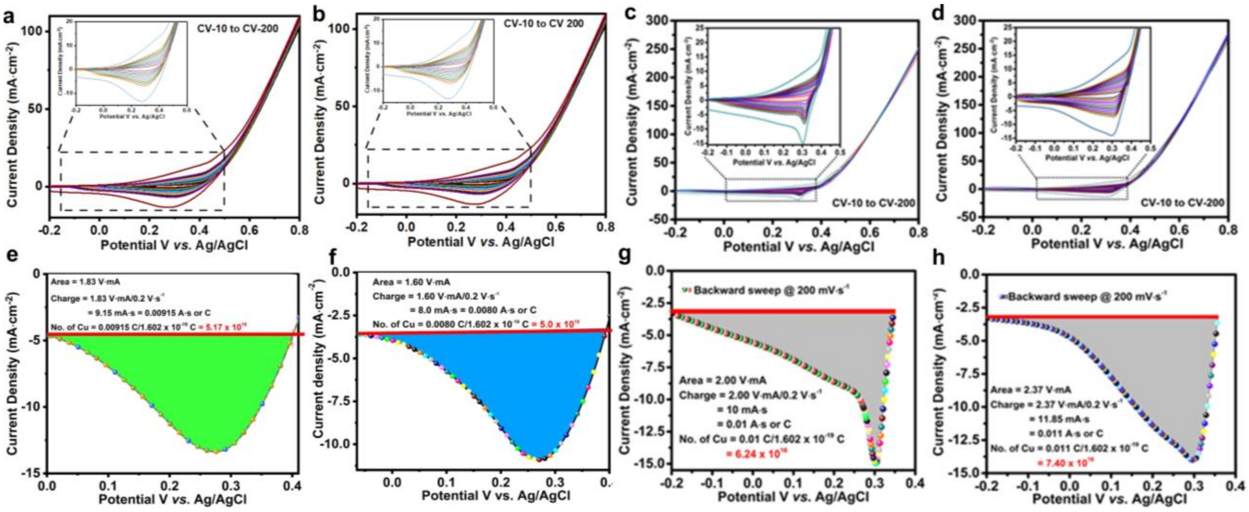

Supplementary Figure 14. a-d) CV curves collected over B-CF and as-prepared CuO/CF in 1M KOH containing 1M MeOH or 1M EtOH at scan rates of 10, 20, 30, 40, 50, 60, 70, 80, 90, 100, and 200 mV s^{-1}. e-h) Charge integration of backward CV curves collected over B-CF and as-prepared CuO/CF for MOR and EOR under similar conditions. The inset in Fig. a-d) shows the scan rate dependence of the Cu^{2+}↔Cu^{3+} redox couple.

7. Supplementary Note 7

Turnover frequency (TOF) was calculated at potentials beyond 1.42 V versus RHE and up to 1.82 V versus RHE with a regular interval of 0.2 V. Equation 4 was used in the calculation of TOF of methanol electro oxidation with CuO/CF.

$$TOF = j \times \frac{NA}{(n \times F \times \Gamma)} \quad (6)$$

In eq 6, j denotes current density, N_A denotes the Avogadro's number, n denotes the number of electrons transferred for the oxidation of a single methanol or ethanol molecule to formate and acetate (four in this case), F denotes the Faraday constant, and Γ denotes the surface concentration or more precisely the number of electrochemically accessible active sites of Cu in CuO/CF.

$$TOF_{Formate} = \frac{FE_{Formate}}{100} \times TOF \quad (7)$$

$$TOF_{Acetate} = \frac{FE_{Acetate}}{100} \times TOF \quad (8)$$

The $TOF_{Formate}$ and $TOF_{Acetate}$ were calculated by using eq 7 and 6. Where $FE_{Formate}$ and $FE_{Acetate}$ are the Faraday efficiencies of *as*-prepared CuO/CF for formate and acetate at the given potential.

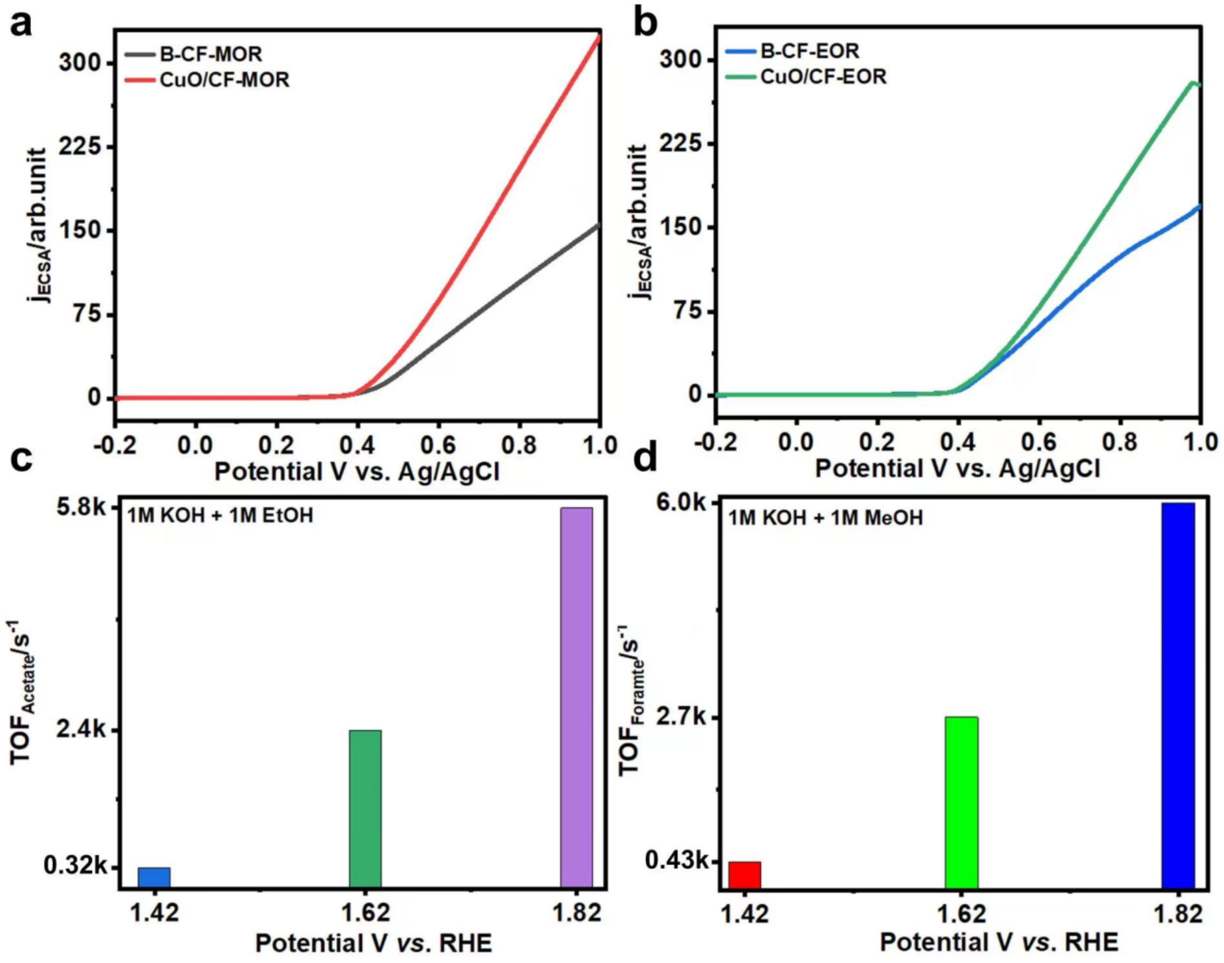

Supplementary Figure 15. Comparison of specific activity for B-CF and as-prepared CuO/CF. a) MOR current densities normalized by the relative *ECAS*. b) EOR current densities normalized by the relative

ECAS. Turnover Frequency (*TOF*) of as-prepared CuO/CF for acetate and formate at given applied potential normalized by their respective Faraday efficiencies. c) $TOF_{Acetate}$ and d) $TOF_{Formate}$.

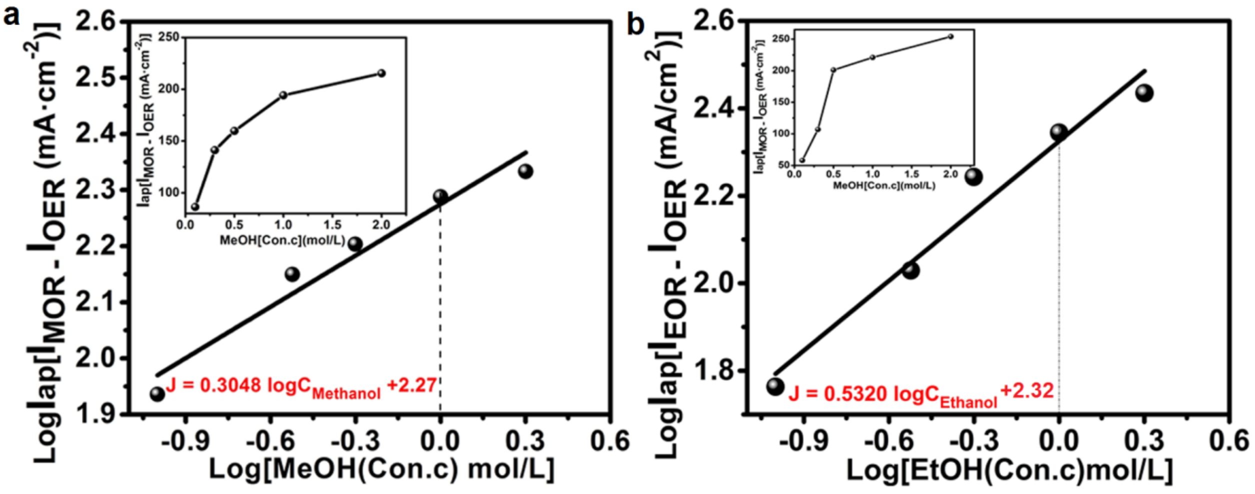

Supplementary Figure 16. a-b) The curve of peak current densities *vs*. concentrations of methanol and ethanol on the as-prepared CuO/CF electrode at 0.8 V *vs*. Ag/AgCl. The inset in Figure (a,b) represents the fitting of methanol and ethanol reaction order in MOR and EOR in 1M KOH.

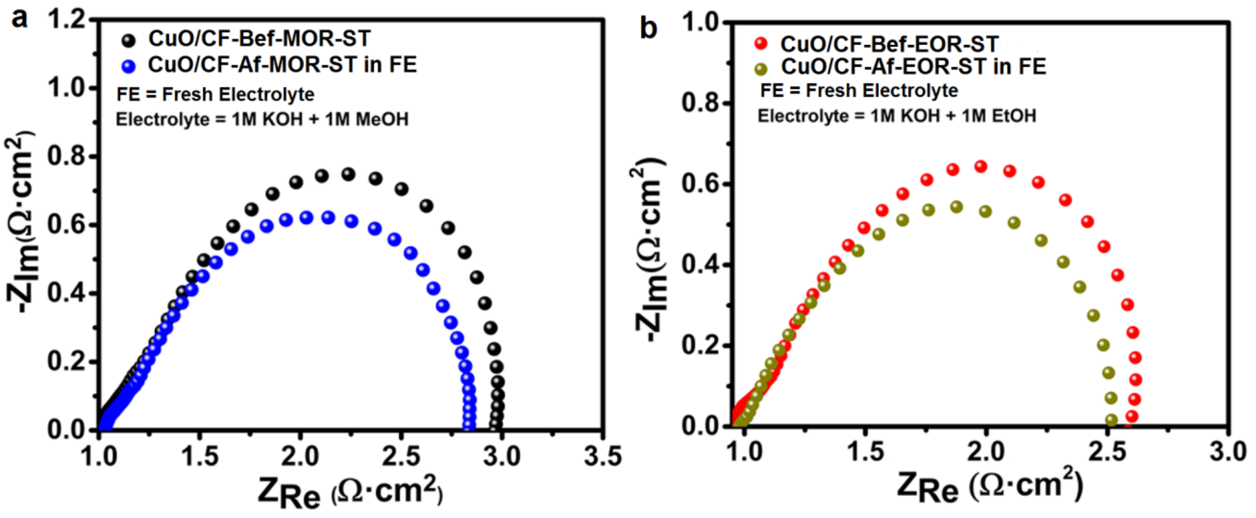

Supplementary Figure 17. a) EIS plot of the as-prepared CuO/CF before (black curve) and after (blue curve) long-term MOR. b) EIS plot of the as-prepared CuO/CF before (red curve) and after (olive green curve) long-term EOR.

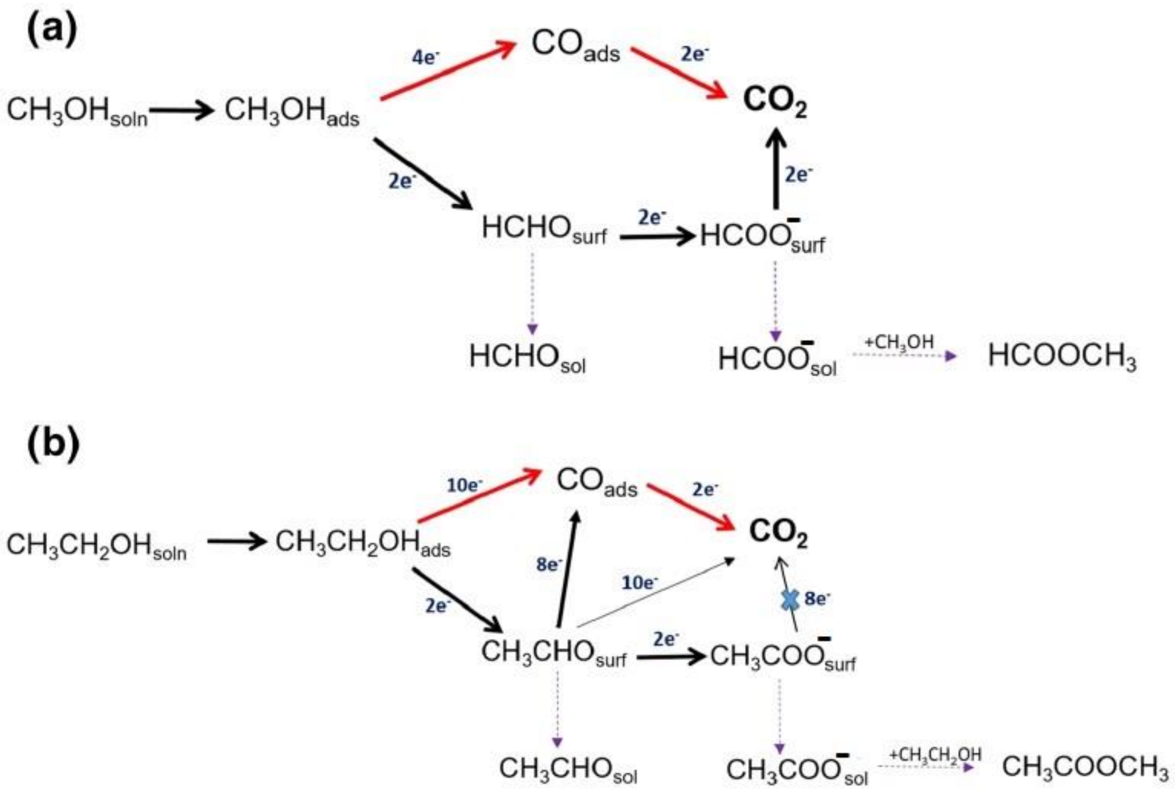

Supplementary Figure 18. a-b) MOR and EOR mechanism in KOH

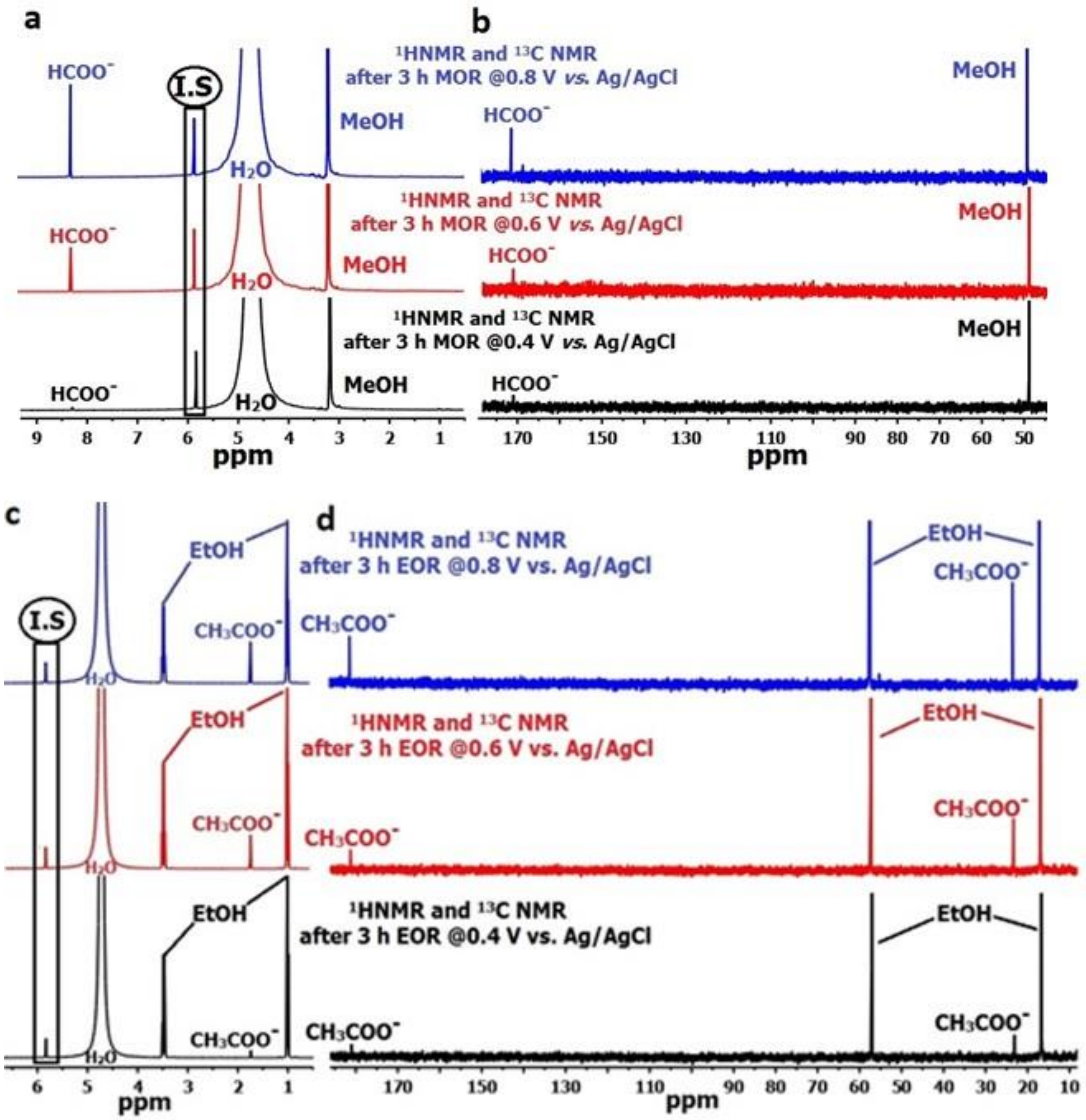

Supplementary Figure 19. a, b) ^{1}HNMR and ^{13}C NMR of the samples collected after 3 hours of MOR at different potentials (0.4, 0.6, and 0.8 V *vs*. Ag/AgCl). c,d) ^{1}HNMR and ^{13}C NMR of the samples collected after 3 hours of EOR at different potentials (0.4, 0.6, and 0.8 V *vs*. Ag/AgCl).

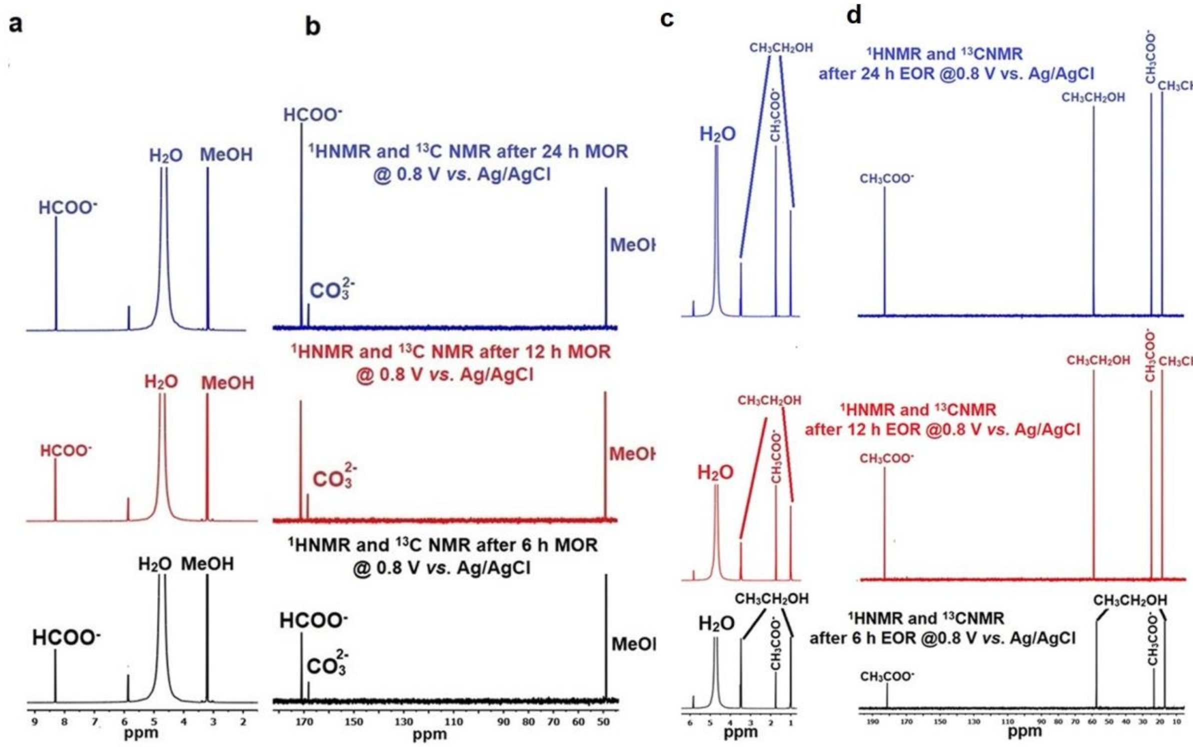

Supplementary Figure 20. a,b) ^{13}C NMR and ^{1}HNMR of the sample collected after different durations of MOR at 0.8V *vs*. Ag/AgCl. c,d) ^{13}C NMR and ^{1}HNMR of the sample collected after different durations of EOR at 0.8V *vs*. Ag/AgCl.

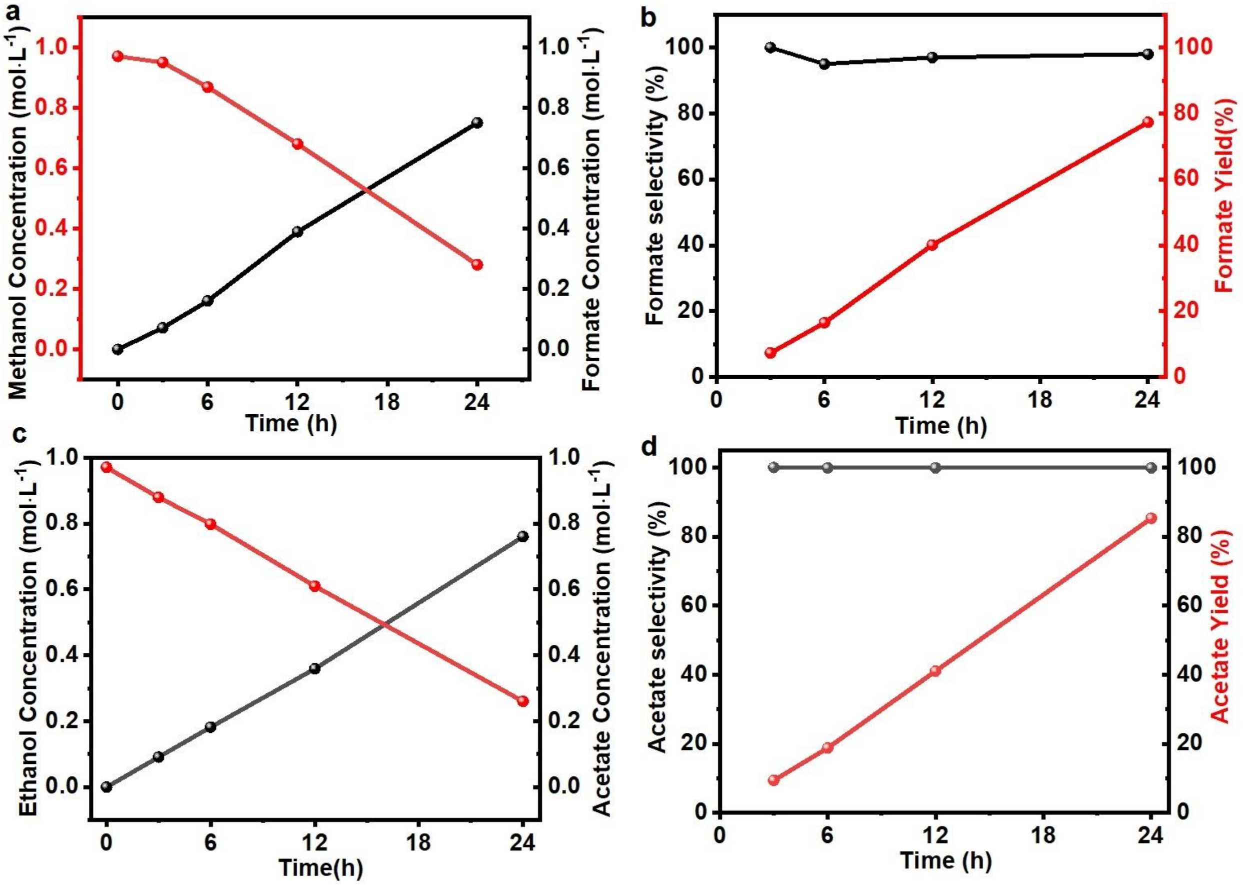

Supplementary Figure 21. a,c) Concentrations of methanol, ethanol, formate, and acetate as a function of time at a constant potential of 1.82 V *vs*. RHE. b,d) Yield and selectivity of formate and acetate as a function of time at a constant potential of 1.82 V *vs*. RHE. Slight volatilization of methanol is inevitable during the experiment.

8. Supplementary Note 8

Supplementary Figure 22a-c displays the effect of carbon monoxide (CO), a reaction intermediate that is formed during the alcohol oxidation reaction. CO is regarded to hinder the electrocatalytic activities of electrocatalyst through the CO poisoning effect. So, it is of prime importance to develop an electrocatalyst with desirable resistance to CO poisoning. To evaluate the effect of CO on the performance of the as-prepared CuO/CF, CO was bubbled in the electrolyte solution for 45 min before and during the experiment. EIS, CV, and long-term stability test in CO saturated electrolyte reveal that the as-prepared CuO/CF is sufficiently active towards CO oxidation as illustrated in Supplementary Figure 22a-c. Remarkably, the peak at 0.44 V *vs*. Ag/AgCl attributed to CuOOH, has become more distinctive during the CO oxidation, clarifying a probable interaction between CuOOH active sites and CO molecules as evident from

Supplementary Figure 22a. The as-prepared CuO/CF exhibits a better MOR CV profile in the presence of CO as compared to the MOR CV in the absence of CO. The oxidation of CO on the surface of the as-prepared CuO/CF occurs through the Eley-Rideal mechanism in which formerly adsorbed O_2 molecules react with CO species. The EIS and long-term experiments were also conducted in the presence and absence of CO. The as-prepared CuO/CF has low R_{Ω}, R_{CT} values in the presence of CO, which indicates the effective charge transfer and fast interfacial kinetics in the presence of CO see Supplementary Figure 22b. The as-prepared CuO/CF robustly catalyzed the CO oxidation for 10000 s as presented in Supplementary Figure 22c. Therefore, these results reflect that the as-prepared CuO/CF exhibits an outstanding resistance toward CO poising.

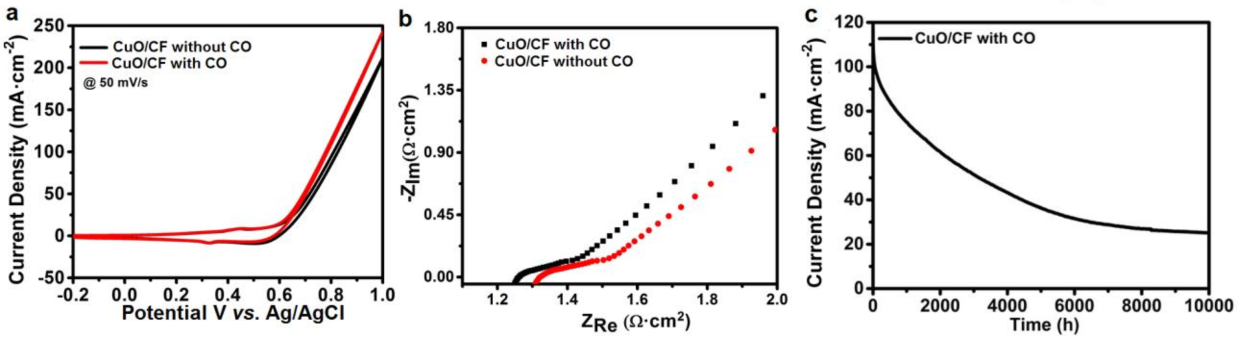

Supplementary Figure 22. a) CV curves collected over the as-prepared CuO/CF in 1M KOH + 1M MeOH in the presence/absence of CO. b) EIS spectra collected over the as-prepared CuO/CF in 1M KOH + 1M MeOH in the presence/absence of CO. c) Long-time oxidation of CO in 1M KOH at 1.82 V *vs*. RHE.

9. Supplementary Table 1.

Redox parameter for selected electrocatalysts in 1M KOH solution[a].

Catalyst	J_{pa} (mA)	E_{pa} (V *vs.* RHE)	J_{pc} (mA)	E_{pc} (V *vs.* RHE)	ΔEp (V)	Γ^* mol·cm^{-2} ($\times 10^{-7}$)	D cm^2·s^{-1} ($\times 10^{-7}$)	Ref
Ni_3C	5.38	1.40	-3.58	1.30	0.13	1.93	0.0599	5
Ni-Co-Se	8.43	1.38	-5.57	1.29	0.10	1.40	0.128	6
Ni-Se	10.8	1.44	-6.9	1.31	0.14	2.1	0.0002	2
$Ni_{97}Bi_3$	29	1.39	-19.0	1.27	0.12	3.97	2	4
Ni-Co-P	4.40	1.38	-2.39	1.25	0.13	-	-	7
B-CF	4.58	0.44	-3.78	0.30	0.14	0.544	0.0261	This work
CuO/CF	8.24	0.42	-7.67	0.26	0.16	1.43	0.214	This work

[a]CuO/CF shows two cathodic/anodic peaks, J_{pa}, J_{pc}, E_{pa}, E_{pc}, and ΔE calculated from the tallest voltage peak. CVs were performed in 1M KOH electrolyte at 10 mV·s^{-1}.

10. Supplementary Table 2.

Comparison of MOR performance between CuO/CF and reported non-noble catalyst

S.N	Catalyst	Morphology	Onset potential V *vs.* RHE	Current density j(mA·cm^{-2}) @V *vs.* RHE geometric MOR	[a] MOR I_{ap} (mA·cm^{-2}) @V *vs.* RHE	Γ^* mol·cm^{-2} (x10^{-7})	D cm^2·s^{-1} (x10^{-7})	Electrolyte	Ref
1	$Ni_{2.5}Co_{0.5}Sn_2$	Nanoparticles	1.38	65.5@1.57	-	1.4	0.128	1M MeOH + 1M KOH	6
2	Ni_3Sn_2	Nanoparticles	-	34.4@1.57	-	0.86	0.084	1M MeOH + 1M KOH	
3	$Ni_{0.75}Cu_{0.25}$	3D Networks	1.35	160@2.0	84@1.69	4.48	0.289	0.5M MeOH + 1M NaOH	8
4	Ni-NP	Nanoparticles		35@2.0	12@1.64	0.89	-	0.4M MeOH + 1M KOH	9
5	NiFe-NP	Nanoparticles	1.40	50@1.56	-	-	-	1M MeOH + 1M NaOH	10
6	NiO/Ni@C NT	Nanostructure	1.43	2.7@1.82	1.5@1.62	-	-	1M MeOH + 1M KOH	11
7	Ni_2Co_2	Cauliflower	-	120@2.0	~35@1.74	1.63	-	0.5M MeOH + 1M NaOH	12
8	$Ni_4(OH)_4$	Porous	-	-	30@1.62	1.63	-	0.5M MeOH + 1M KOH	13
9	Ni/Ti	Flakes	-	39@1.62	-	-	-	0.5M MeOH + 1.0M NaOH	14

10	Ni-Ti-NP	Nanoparticles	-	0.65@2.0	0.5@1.62	-	-	0.1M NaOH + 0.2MeOH	15
11	NiMn	Film	-	115@2.0	80@1.64	0.62	-	0.5M MeOH + 1M NaOH	16
12	$Ni_{1.7}Sn$	Nanoparticles	1.41	80@2.0	51@1.65	2.1	0.00019	0.5M MeOH + 0.5M KOH	2
13	Ni/SBACPE -NP	Mesoporous	-	23@2.0	14.2@1.8	0.90	-	0.03M MeOH + 0.1M NaOH	17
14	CuNi	Porous NWs	-	-	21@1.85	-	-	0.2M MeOH + 0.1M NaOH	18
15	$NiCo_2O_4$/G CE	Nanoflakes	-	15.5@2.0	11@1.67	-	-	1M MeOH + 0.1M NaOH	19
16	Cu/NiCu-NW	Nanowires	1.25	34.9@1.55	-	0.026	-	1M MeOH + 1M KOH	20
17	NiO-NS@NW	Nanowires	-	89@1.62	-	-	-	0.5 M MeOH + 1M KOH	21
18	$Ni_{0.2}Co_{0.2}$-G	Nanoparticles	-	225@2.0	-	3.05	-	2M MeOH + 1M KOH	22
19	$NiMoO_4$/C	Nanorods	-	49@1.79	-		-	2M MeOH + 1M KOH	23
20	Ni-beta-SDS/GC	Zeolite	-	21@1.76	-	-	-	0.1M MeOH + 0.1M KOH	24
21	$Ni_{97}Bi_3$ aerogel	Nanowires	1.33	199@1.66	-	3.97		1M MeOH + 1M KOH	4
22	NiCo/NiO-CoO	Petal-like	1.34	178@1.65	-	-	-	0.5M MeOH + 0.5M KOH	25
23	NiO-NS	Nanosheets	1.37	85.3@1.72		-	-	0.5M MeOH + 1M KOH	26
24	Mn-$Ni(OH)_2$	Hollow	1.35	23@2.0	16.7@1.6	-	-	0.5M MeOH + 1M KOH	27
25	NiO-NTs-400	Nanotubes	1.33	24.3@1.50	-	3.75	-	0.5M MeOH + 1M NaOH	28
26	KB&CTGU -15	MOFs	1.43	29.8@1.62	-	-		1M MeOH + 0.1M KOH	13
28	Cu@CoO_x	Core-cage	1.33	305.4@1.88	150@1.88	-	-	1M MeOH + 1M NaOH	29
29	CuO/$Co(OH)_2$	Nanosheets	1.35	-	159@1.65	-	-	3M MeOH + 1M NaOH	30
30	$Cu(OH)_2$ CuO/Cu	Needle Like	-	42@1.52	-	-	-	0.5M MeOH + 0.5M KOH	31
31	CuO	Granular	-	10@1.52	-	-	-	0.5M MeOH + 0.5M KOH	32
32	CuS	Microflower	-	3@1.52	-	-	-	0.5M MeOH + 0.5M KOH	33
33	$Cu(OH)_2$@$CoCO_3(OH)_2\cdot nH_2O$	Nanowire	-	78@1.52	-	-	-	0.5M MeOH + 0.5M KOH	34
34	$Ni_1Co_2P_x$	Porous	-	155@1.7				1M MeOH + 1M KOH	7

35	$Ni_{0.75}Fe_{0.25}Se_2$	Nanoparticles	-	50@1.5	-	0.73	0.0022	0.5M MeOH + 1M KOH	[35]
36	MoS_2@CoNi-ZIF	Nanoparticles	1.43	60@1.6	-	-	-	0.5M MeOH + 1M KOH	[28]
37	$Co_3O_4/NiCo_2O_4$	Porous	1.38	140@1.64	-	-	-	0.5M MeOH + 1M KOH	[36]
38	NiO	Nanosheets	-	85.3@1.72	-	-	-	0.5M MeOH + 1M KOH	[26]
39	NiFe-LDH	Nanosheets	-	25@1.45	-	-	-	0.5M MeOH + 1M KOH	[37]
40	CuO/CF	Nanosheets array	1.34	100@1.71	86@1.75	1.43	0.214	0.1M MeOH + 1M KOH	This Work
				200@1.94					
				100@1.65	141@1.84			0.3M MeOH + 1M KOH	
				200@1.82					
				100@1.64	159@1.85			0.5M MeOH + 1M KOH	
				200@1.79					
				100@1.61	194@1.86			1M MeOH + 1M KOH	
				200@1.75					
				100@1.58	215@1.86			2M MeOH + 1M KOH	
				200@1.72					

[a] Values calculated by subtracting the corresponding baseline current obtained in the absence of methanol

11. Supplementary Table 3.

Comparison of EOR performance between CuO/CF and reported non-noble catalysts.

S.N	Catalyst	Morphology	Onset potential V *vs.* RHE	Current density j(mA·cm^{-2}) @V *vs.* RHE geometric MOR	[a] MOR I_{ap} (mA·cm^{-2}) @V *vs.* RHE	Γ^* mol·cm^{-2} (x10^{-7})	*D* cm^2·s^{-1} (x10^{-7})	Electrolyte	Ref
1	NiO/HC	Nanoparticles	0.45	55@1.72	28.5@1.59		-	1.0 EtOH + 1.0M NaOH	[38]
3	Ni_xCo_{1-x}/CNF	Nanoparticles	-	142@1.77	-	-	-	2M EtOH + 1M KOH	[39]
4	$Co_{0.2}Ni_{0.2}$	Nanoparticles	0.38	75@1.62	-		-	1EtOH + 1M KOH	[40]
5	Ni-B-NT	Nanotubes	-	30@1.72	19@0.6	-	-	0.5M EtOH + 0.1M NaOH	[41]
6	NiNC-3	Nanorods	0.34	71@1.64	-	-	-	1M EtOH + 0.1M KOH	[42]
7	NGr-NiO/Pulse	Nanoparticles	-	2.3	-	-	-	1M EtOH + 0.5M NaOH	[43]
8	TiO_2/ZnO/H	Nanostructure	-	25@2	-	-	-	1M EtOH + 1M KOH	[44]
9	NiO@C/CC	Nanoparticles	-	119@2	125@1.81	-	-	0.5EtOH + 1M NaOH	[45]
10	Au/MnO_2	Grass-like		63@1.6	-	-		0.5EtOH + 0.5M KOH	[46]
11	CoNi-PHNs	Nanosheets	-	50@1.48	-	--		1M EtOH + 1M KOH	[47]
12	NiFe-LDH	Nanosheets	-	25@1.4	-	--		1M EtOH + 1M KOH	[37]
13	CuO/CF	Nanosheets array	1.34	100@1.84<br>200@1.94	58@1.7	1.43	0.214	0.1M EtOH + 1M KOH	This Work
				100@1.73<br>200@1.82	105@1.8			0.3M EtOH + 1M KOH	
				100@1.61<br>200@1.79	201@1.87			0.5M EtOH + 1M KOH	
				100@1.59<br>200@1.72	221@1.88			1M EtOH + 1M KOH	
				100@1.56<br>200@1.72	254@1.88			2M EtOH + 1M KOH	

[a] Values calculated by subtracting the corresponding baseline current obtained in the absence of ethanol.

12. Supplementary References

1. Cao C, Ma DD, Jia J, Xu Q, Wu XT, Zhu QL. Divergent Paths, Same Goal: A Pair-Electrosynthesis Tactic for Cost-Efficient and Exclusive Formate Production by Metal-Organic-Framework-Derived 2D Electrocatalysts. *Adv. Mater.* **33**, 2008631 (2021).

2. Li J, *et al.* NiSn bimetallic nanoparticles as stable electrocatalysts for methanol oxidation reaction. *Appl. Catal. B* **234**, 10-18 (2018).

3. Zhang S-J, Zheng Y-X, Yuan L-S, Zhao L-H. Ni–B amorphous alloy nanoparticles modified nanoporous Cu toward ethanol oxidation in alkaline medium. *J. Power Sources* **247**, 428-436 (2014).

4. Dubale AA, *et al.* High-Performance Bismuth-Doped Nickel Aerogel Electrocatalyst for the Methanol Oxidation Reaction. *Angew. Chem. Int. Ed.* **59**, 13891-13899 (2020).

5. Li J, *et al.* Selective Methanol-to-Formate Electrocatalytic Conversion on Branched Nickel Carbide. *Angew. Chem. Int. Ed.* **59**, 20826-20830 (2020).

6. Li J, *et al.* Colloidal Ni–Co–Sn nanoparticles as efficient electrocatalysts for the methanol oxidation reaction. *J. Mater. Chem. A* **6**, 22915-22924 (2018).

7. Chen S, *et al.* Design of 3D Hollow Porous Heterogeneous Nickel–Cobalt Phosphides for Synergistically Enhancing Catalytic Performance for Electrooxidation of Methanol. *ACS Appl. Mater. Interfaces* **12**, 34971-34979 (2020).

8. Cui X, *et al.* Highly Branched Metal Alloy Networks with Superior Activities for the Methanol Oxidation Reaction. *Angew. Chem. Int. Ed.* **56**, 4488-4493 (2017).

9. Abdel Hameed RM, El-Sherif RM. Microwave irradiated nickel nanoparticles on Vulcan XC-72R carbon black for methanol oxidation reaction in KOH solution. *Appl. Catal. B Environ.* **162**, 217-226 (2015).

10. Candelaria SL, *et al.* Multi-Component Fe–Ni Hydroxide Nanocatalyst for Oxygen Evolution and Methanol Oxidation Reactions under Alkaline Conditions. *ACS Catal.* **7**, 365-379 (2017).

11. Wang J, *et al.* Fabrication of nanoscale NiO/Ni heterostructures as electrocatalysts for efficient methanol oxidation. *J. Mater. Chem. A* **5**, 9946-9951 (2017).

12. Cui X, *et al.* Promoting effect of Co in Ni_mCo_n (m + n = 4) bimetallic electrocatalysts for methanol oxidation reaction. *ACS Appl. Mater. Interfaces* **7**, 493-503 (2015).

13. Wu YP, *et al.* Bi-Microporous Metal-Organic Frameworks with Cubane $[M_4(OH)_4]$ (M=Ni, Co) Clusters and Pore-Space Partition for Electrocatalytic Methanol Oxidation Reaction. *Angew. Chem., Int. Ed.* **58**, 12185-12189 (2019).

14. Yi Q, Huang W, Zhang J, Liu X, Li L. Methanol oxidation on titanium-supported nano-scale Ni flakes. *Catal. Commun.* **9**, 2053-2058 (2008).

15. Yu Y, Yang Q, Li X, Guo M, Hu J. A bimetallic Ni–Ti nanoparticle modified indium tin oxide electrode fabricated by the ion implantation method for studying the direct electrocatalytic oxidation of methanol. *Green Chem.* **18**, 2827-2833 (2016).

16. Danaee I, Jafarian M, Mirzapoor A, Gobal F, Mahjani M. Electrooxidation of methanol on NiMn alloy modified graphite electrode. *Electrochim. Acta* **55**, 2093-2100 (2010).

17. Azizi SN, Ghasemi S, Chiani E. Nickel/mesoporous silica (SBA-15) modified electrode: An effective porous material for electrooxidation of methanol. *Electrochim. Acta* **88**, 463-472 (2013).

18. Ding R, Liu J, Jiang J, Wu F, Zhu J, Huang X. Tailored Ni–Cu alloy hierarchical porous nanowire as a potential efficient catalyst for DMFCs. *Catal. Sci. Technol.* **1**, 1406-1411 (2011).

19. Anu Prathap MU, Srivastava R. Synthesis of $NiCo_2O_4$ and its application in the electrocatalytic oxidation of methanol. *Nano Energy* **2**, 1046-1053 (2013).

20. Wu D, Zhang W, Cheng D. Facile Synthesis of Cu/NiCu Electrocatalysts Integrating Alloy, Core–Shell, and One-Dimensional Structures for Efficient Methanol Oxidation Reaction. *ACS Appl. Mater. Interfaces* **9**, 19843-19851 (2017).

21. Luo Q, Peng M, Sun X, Asiri AM. Hierarchical nickel oxide nanosheet@nanowire arrays on nickel foam: an efficient 3D electrode for methanol electro-oxidation. *Catal. Sci. Technol.* **6**, 1157-1161 (2016).

22. Barakat NAM, Motlak M. Co_xNi_y-decorated graphene as novel, stable and super effective non-precious electro-catalyst for methanol oxidation. *Appl. Catal. B* **154-155**, 221-231 (2014).

23. Jothi PR, Kannan S, G V. Enhanced methanol electro-oxidation over in-situ carbon and graphene supported one dimensional $NiMoO_4$ nanorods. *J. Power Sources* **277**, 350-359 (2015).

24. Liao Y, Pan S, Bian C, Meng X, Xiao F-S. Improved catalytic activity in methanol electro-oxidation over the nickel form of aluminum-rich beta-SDS zeolite modified electrode. *J. Mater. Chem. A* **3**, 5811-5814 (2015).

25. Rezaee S, Shahrokhian S. Facile synthesis of petal-like NiCo/NiO-CoO/nanoporous carbon composite based on mixed-metallic MOFs and their application for electrocatalytic oxidation of methanol. *Appl. Catal. B* **244**, 802-813 (2019).

26. Yang W*, et al.* Oxygen vacancies confined in ultrathin nickel oxide nanosheets for enhanced electrocatalytic methanol oxidation. *Appl. Catal. B* **244**, 1096-1102 (2019).

27. Dong B*, et al.* Fabrication of hierarchical hollow Mn doped $Ni(OH)_2$ nanostructures with enhanced catalytic activity towards electrochemical oxidation of methanol. *Nano Energy* **55**, 37-41 (2019).

28. Liu Y*, et al.* Hierarchical nanocomposite electrocatalyst of bimetallic zeolitic imidazolate framework and MoS_2 sheets for non-Pt methanol oxidation and water splitting. *Appl. Catal. B* **258**, 117970 (2019).

29. Sun Y*, et al.* Synergistic Cu@CoO_x core-cage structure on carbon layers as highly active and durable electrocatalysts for methanol oxidation. *Appl. Catal. B* **244**, 795-801 (2019).

30. Chen L, Hua Z, Shi J, He M. CuO/$Co(OH)_2$ Nanosheets: A Novel Kind of Electrocatalyst for Highly Efficient Electrochemical Oxidation of Methanol. *ACS Appl. Mater. Interfaces* **10**, 39002-39008 (2018).

31. Anantharaj S, Sugime H, Noda S. Ultrafast Growth of a $Cu(OH)_2$-CuO Nanoneedle Array on Cu Foil for Methanol Oxidation Electrocatalysis. *ACS Appl. Mater. Interfaces* **12**, 27327-27338 (2020).

32. Pawar SM*, et al.* Multi-functional reactively-sputtered copper oxide electrodes for supercapacitor and electro-catalyst in direct methanol fuel cell applications. *Sci. Rep.* **6**, 21310 (2016).

33. Radhakrishnan S, Kim H-Y, Kim B-S. Expeditious and eco-friendly fabrication of highly uniform microflower superstructures and their applications in highly durable methanol oxidation and high-performance supercapacitors. *J. Mater. Chem. A* **4**, 12253-12262 (2016).

34. Xie L, Tang C, Wang K, Du G, Asiri AM, Sun X. $Cu(OH)_2@CoCO_3(OH)_2 \cdot nH_2O$ Core–Shell Heterostructure Nanowire Array: An Efficient 3D Anodic Catalyst for Oxygen Evolution and Methanol Electrooxidation. *Small* **13**, 1602755 (2017).

35. Li J, *et al.* Nickel Iron Diselenide for Highly Efficient and Selective Electrocatalytic Conversion of Methanol to Formate. *Small* **17**, 2006623 (2021).

36. Qian L, Luo S, Wu L, Hu X, Chen W, Wang X. In situ growth of metal organic frameworks derived hierarchical hollow porous $Co_3O_4/NiCo_2O_4$ nanocomposites on nickel foam as self-supported flexible electrode for methanol electrocatalytic oxidation. *Appl. Surf. Sci.* **503**, 144306 (2020).

37. Mondal B, *et al.* Unraveling the Mechanisms of Electrocatalytic Oxygenation and Dehydrogenation of Organic Molecules to Value‐Added Chemicals Over a Ni–Fe Oxide Catalyst. *Adv. Energy Mater.* **11**, 2101858 (2021).

38. Cuña A, *et al.* Electrochemical and spectroelectrochemical analyses of hydrothermal carbon supported nickel electrocatalyst for ethanol electro-oxidation in alkaline medium. *Appl. Catal. B* **202**, 95-103 (2017).

39. Barakat NAM, Motlak M, Elzatahry AA, Khalil KA, Abdelghani EAM. Ni_xCo_{1-x} alloy nanoparticle-doped carbon nanofibers as effective non-precious catalyst for ethanol oxidation. *Int. J. Hydrogen Energy* **39**, 305-316 (2014).

40. Barakat NAM, Motlak M, Lim BH, El-Newehy MH, Al-Deyab SS. Effective and Stable CoNi Alloy-Loaded Graphene for Ethanol Oxidation in Alkaline Medium. *J. Electrochem. Soc.* **161**, F1194-F1201 (2014).

41. Muench F, *et al.* Electroless synthesis of nanostructured nickel and nickel–boron tubes and their performance as unsupported ethanol electrooxidation catalysts. *J. Power Sources* **222**, 243-252 (2013).

42. Shi W, *et al.* One-step synthesis of N-doped activated carbon with controllable Ni nanorods for ethanol oxidation. *Electrochim. Acta* **220**, 486-492 (2016).

43. Daryakenari AA, Hosseini D, Mirfasih MH, Apostoluk A, Müller CR, Delaunay J-J. Formation of NiO nanoparticle-attached nanographitic flake layers deposited by pulsed electrophoretic deposition for ethanol electro-oxidation. *J. Alloys Compd.* **698**, 571-576 (2017).

44. Tolba GMK, *et al.* Hierarchical TiO_2/ZnO Nanostructure as Novel Non-precious Electrocatalyst for Ethanol Electrooxidation. *J. Mater. Sci. Technol.* **31**, 97-105 (2015).

45. Liu C, *et al.* Air-Assisted Transient Synthesis of Metastable Nickel Oxide Boosting Alkaline Fuel Oxidation Reaction. *Adv. Energy Mater.* **10**, 2001397 (2020).

46. Bigiani L, *et al.* Engineering Au/MnO_2 hierarchical nanoarchitectures for ethanol electrochemical valorization. *J. Mater. chem. A* **8**, 16902-16907 (2020).

47. Wang W, *et al.* Modulation of Molecular Spatial Distribution and Chemisorption with Perforated Nanosheets for Ethanol Electro-oxidation. *Adv. Mater.* **31**, 1900528 (2019).
